# House dust mite and Cockroach specific Immunoglobulin E sensitization is associated with diabetes mellitus in the adult Korean population

**DOI:** 10.1038/s41598-018-20573-0

**Published:** 2018-02-08

**Authors:** Mee Kyoung Kim, Jee Sun Jeong, Kyungdo Han, Ki Hyun Baek, Ki-Ho Song, Hyuk-Sang Kwon

**Affiliations:** 1 Department of Internal Medicine, College of Medicine, The Catholic University of Korea, Seoul, Korea; 2 Department of Medical Statistics, College of Medicine, The Catholic University of Korea, Seoul, Korea

## Abstract

Immunoglobulin E (IgE) is known to activate mast cells. Prior studies have shown that mast cells contribute to diet-induced obesity and diabetes mellitus (DM). We aimed to determine whether adults with IgE sensitization were at risk of DM. We performed assays regarding serum total IgE and allergen-specific IgE levels against the house dust mite, the cockroach, and the dog on 1,528 adults randomly sampled from every age and gender group in various districts. The total and three allergen-specific IgE levels were positively correlated with fasting glucose level and insulin resistance. Subjects with increased levels of total IgE (>100 kU/L), compared to those without, had an odds ratio (OR) of 1.72 (95% confidence interval [95% CI], 1.17–2.54) for DM after adjusting for various covariates. Further controlling for previous allergic disease did not attenuate the association between total IgE level and DM. Subjects sensitized to the house dust mite (OR 1.63, 95% CI, 1.03–2.59) and the cockroach (OR 2.27, 95% CI, 1.40–3.66) were also at increased risk of DM. We found a strong positive association between IgE sensitization and DM in a general Korean population, suggesting that IgE may be an important independent risk factor for metabolic diseases in Koreans.

## Introduction

Prior studies have shown that mast cells contribute to diet-induced obesity and diabetes mellitus (DM)^1,2^. Inflammatory mediators released by mast cells increase capillary permeability and trigger vasoconstriction and endothelial cell remodeling in patients with atherosclerosis^3–5^. Moreover, these mediators increase cytokine-induced insulin resistance (IR) and impair insulin secretion^2^. Mice lacking mast cells or given the mast cell inhibitors cromolyn or ketotifen are fully protected from the development of type 2 DM^1^. In animal models, mast cells participate directly in the development of diet-induced obesity and diabetes, and mast cell inhibitors offer hope to patients with these common, chronic inflammatory conditions^1^.

Immunoglobulin E (IgE) antibody binds to the fragment crystallizable (Fc) receptors located principally on mast cell surfaces^6^. IgE plays a key role in the signaling response to allergens. The binding of IgE to an Fc receptor activates mast cell degranulation and the release of cytokines, chemokines, histamine, proteoglycan, and mast cell protease. Clinically, IgE levels are increased in patients with atopic dermatitis (AD), asthma, and hay fever^7^. Recently, IgE levels have been shown to be elevated in patients with several chronic diseases, including rheumatoid arthritis, atherosclerosis, and ischemic heart disease^3^. Importantly, a recent US study found a higher prevalence of type 2 DM in patients with atopic dermatitis (AD) than in the general population^7^. Possible explanations include shared genetic risk loci for AD and type 2 DM^8^; diabetogenic effects of chronic, systemic low-grade inflammation in patients with moderate-to-severe AD^9^; and/or a sedentary lifestyle associated with AD^7,10^. We hypothesized that IgE sensitization might play a role in the development of type 2 DM. In the present study, we analyzed nationally representative data from the Korean National Health and Nutrition Examination Survey (KNHANES) to explore whether adults with IgE sensitization were at an increased risk of DM independent of allergic disease status. We also examined the relationship between IgE sensitization and metabolic syndrome (insulin resistance syndrome).

## Methods

**Figure 1:**
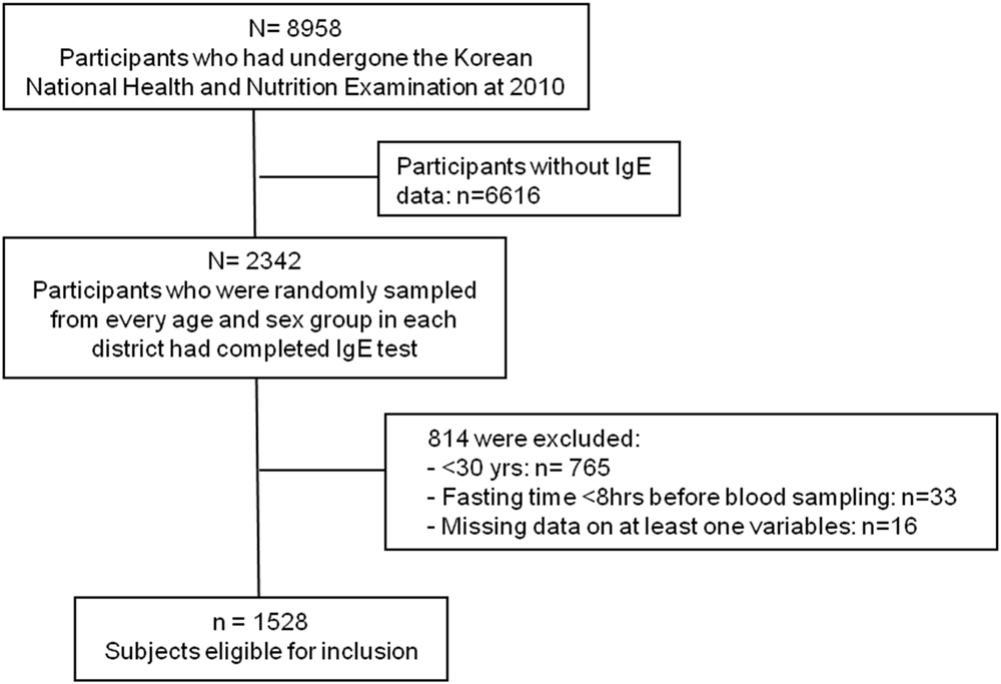
Flow chart of the study population.

We used data acquired during the Korean National Health and Nutrition Examination Survey (KNHANES), which is conducted by the Korea Center for Disease Control and Prevention to obtain nationally representative and reliable statistical data on the health, health behaviors, nutrition, and food intake of the Korean population. KNHANES surveys are conducted annually using a rolling sample design featuring a complex, stratified, multistage probability-cluster survey of a representative sample of the non-institutionalized civilian population of South Korea^11–13^. The survey was performed by the Korean Ministry of Health and Welfare and had three components: a health interview survey, a health examination survey, and a nutrition survey. Notably, the 2010 KNHANES gathered information on serum total and allergen-specific IgE levels from 2,342 participants randomly chosen from every age and sex group in each district; thus, the data were representative of the general Korean population^11,12^. Of the 2,342 participants, we excluded those younger than 30 years (n = 765); those who had not fasted sufficiently prior to blood sampling (<8 h) (n = 33); and those for whom data on at least one variable were missing (n = 16). Ultimately, the study population consisted of 1,528 subjects (Fig. 1). The Korea Center for Disease Control and Prevention (KCDC) Institutional Reviews Board approved all survey protocols (numbers 2010–02CON-21-C), and participants provided informed consent before partaking in the study, which was conducted in accordance with the ethical principles of the Declaration of Helsinki.

### Anthropometric measurements

The health interviews and health behavior surveys included well-established questions exploring demographic and socioeconomic characteristics. Anthropometric measurements were performed by specially trained examiners. Body weight and height, which were measured with the subject barefoot and wearing light clothing, were used to calculate the body mass index (BMI). Waist circumference was measured to the nearest 0.1 cm in the horizontal plane at the level of the midpoint between the iliac crest and the costal margin at the end of a normal expiration. Blood pressure was measured three times on the right arm using a mercury sphygmomanometer (Baumanometer; Baum, Copiague, NY, USA) with the individual in a seated position after at least 5 min of rest. The final blood pressure was the average of the second and third measurements.

### Measurement of serum IgE

Fasting blood samples were collected, processed, immediately refrigerated, and transported on ice to the Central Testing Institute in Seoul, Korea. All blood samples were analyzed within 24 h of delivery. Total IgE and allergen-specific IgE levels were measured using an immunoradiometric assay (ImmunoCAP 100, Phadia, Uppsala, Sweden) and a 1470 Wizard gamma-counter (PerkinElmer, Turku, Finland). In Korea, house dust mite, cockroach, mugwort, oak, Japanese hop, ragweed, and dog dander are important inhalant allergens^13^. House dust mite is the primary inhalant allergen, and cockroach is the second leading allergen. Three common indoor allergens were tested for house dust mite (*Dermatophagoides farina*), cockroach and dog. Those with total IgE levels >100 kU/L were categorized as having “increased IgE”^14,15^, and those with allergen-specific IgE levels ≥0.35 kU/L were considered sensitized^11^.

Allergic diseases were defined subjects having one or more of the following disorders; atopic dermatitis (AD), asthma or allergic rhinitis. The following question was used to assess physician-diagnosed AD for each participant: “Have you been diagnosed with AD by a doctor?” Physician-diagnosed asthma and allergic rhinitis were also determined by using similar questions^11^.

### Definitions of DM and metabolic syndrome

DM was defined by a fasting blood glucose (FBG) level ≥126 mg/dL and/or a self-report of physician diagnosis and/or the use of an antidiabetic drug. FBG concentrations were measured using an automated enzymatic assay (Hitachi, Tokyo, Japan); hemoglobin A1C (HbA1C) levels were measured with the aid of high-performance liquid chromatography (HLC-723G7: Tosoh, Yamaguchi, Japan). HbA1C levels were measured in subjects with FBG levels ≥126 mg/dL and those taking insulin or oral anti-diabetic medications. Poor glycemic control was defined as an HbA1c level ≥7%^16^. The homeostasis model assessment of insulin resistance (HOMA-IR) score was calculated as follows: [fasting glucose (mmol/L) × fasting insulin (mIU/L)]/22.5^17^. Subjects were considered to exhibit IR if their HOMA-IR scores were in the highest quartile.

Metabolic syndrome (MetS) was diagnosed when a subject met at least three of the following criteria^18^: (1) waist circumference ≥90 cm in males and ≥ 80 cm in females (using the International Obesity Task Force criteria for the Asia–Pacific region); (2) systolic blood pressure (BP) and/or diastolic BP ≥130/85 mmHg or present antihypertensive drug therapy or a history of hypertension; (3) FBG level ≥100 mg/dL or current medication regimen to treat elevated glucose; (4) fasting serum triglyceride ≥150 mg/dL or current relevant medication regimen; and (5) HDL cholesterol <40 mg/dL in males and <50 mg/dL in females or current relevant medication.

### Statistical analysis

All data are presented as means ± standard errors (SEs) unless stated otherwise. If necessary, logarithmic transformations were performed to normalize distributions. Relationships between participant characteristics and DM were sought using the Student’s *t*-test to compare continuous measures and the χ^2^ test to compare categorical measures. To estimate odds ratios (ORs) for DM by total and allergen-specific (house dust mite, cockroach, and dog) IgE levels, we performed both simple and multiple logistic regression analyses using a generalized linear model appropriate for evaluation of complex survey data. In multivariate analysis, adjusted ORs and 95% confidence intervals (CIs) were calculated after adjustment for age, sex, BMI, educational level, household income, and region of residence (model 1); after adjustment for model 1 plus smoking status, alcohol consumption, and regular exercise (model 2); after adjustment for models 2 plus previous allergic disease status (model 3); and after adjustment for model 3 plus white blood cell counts (model 4). Finally, we added “increased total IgE” and “sensitization of specific allergens” as separate variables in the same multivariate model (model 5). Similar analyses were performed to estimate ORs for poor glycemic control among DM patients (n = 153). All statistical analyses were performed with the aid of SAS version 9.4 software (SAS Institute Inc., Cary, NC, USA). P value < 0.05 was considered to indicate statistical significance and was not adjusted for multiple comparisons.

## Results

### General characteristics of the study population

The cross-sectional analyses included data on 1,528 adults (755 males and 733 females) with a mean age of 49 years; 153 (10%) had DM, and this group was more likely than others to be older, male, and obese and to have a lower income, an educational level less than high school and higher white blood cell (WBC) counts (Table 1). The mean total IgE level and the cockroach sensitization rate were significantly higher in DM subjects (Table 1) (P < 0.001).

**Table 1:** Baseline characteristics of the study subjects by presence or absence of diabetes mellitus.

	Diabetes Mellitus		P-value
	No	Yes	
N	1375	153	
Age, yrs	48.7 ± 0.5	59.8 ± 1.5	**<0.001**
Sex (male), %	47.1 (1.1)	60.3 (5.3)	**0.0221**
BMI, kg/m^2^	23.9 ± 0.1	25.4 ± 0.4	**<0.001**
Waist Circumference, cm	81.6 ± 0.3	87.4 ± 0.9	**<0.001**
Region (urban area), %	76.8 (3.4)	80.5 (5)	0.3766
Income (low 10%), %	19.1 (1.8)	33.9 (5.6)	**<0.001**
Education (>high school), %	65.1 (1.7)	44.5 (5.2)	<0.001
Regular exercise (yes), %	24.2 (1.5)	19.6 (4.5)	0.3668
Ever-smoker (yes), %	44 (1.3)	55.2 (5)	**0.0364**
Systolic BP, mmHg	121.7 ± 0.5	129.3 ± 2	**<0.001**
Diastolic BP, mmHg	78.8 ± 0.4	78.5 ± 0.6	0.6321
Fasting blood sugar, mg/dL	93.6 ± 0.3	145.3 ± 6.0	**<0.001**
Total cholesterol, mg/dL	178.3 ± 1.2	185.5 ± 1.5	<0.001
Triglyceride*, mg/dL	114.9 (111, 119)	161.0 (139, 186)	**<0.001**
HDL cholesterol, mg/dL	52.8 ± 0.4	46.7 ± 1.2	**<0.001**
HOMA-IR*	2.22 (2.16, 2.29)	3.69 (3.26, 4.17)	**<0.001**
White blood cell counts*, 10^3^/μl	5.90 (5.78,6.01)	6.97 (6.44.7.53)	**<0.001**
Total Immunglobulin E*, U/mL	87.6 (79.9, 96.1)	150.4 (114.8, 196.9)	**<0.001**
Increased total IgE, %	43.6 (1.7)	61.9 (4.6)	**<0.001**
Sensitization to House dust mite, %	34.7 (1.4)	41.7 (5.1)	0.1751
Sensitization to cockroaches, %	20.4 (1.4)	37.4 (5.8)	**<0.001**
Sensitization to dog, %	5.1 (0.7)	6 (2.7)	0.7396
Previous allergic diseases ^a^, %	11.8 (1.2)	6.5 (2.3)	0.106

Data are expressed as means ± SE or % (SE). The P-value was calculated by the Student’s *t*-test to compare continuous measures and the χ^2^ test to compare categorical measures.

*Indicates the parameters normalized by log transformation.

^a^Allergic diseases were defined subjects having one or more of the following disorders; eczema (atopic dermatitis), asthma or allergic rhinitis.

BMI, body mass index; BP, blood pressure; HDL, high-density lipoprotein; HOMA-IR, homeostasis model assessment of insulin resistance.

### Correlation between serum IgE levels and metabolic parameters

Pearson’s correlation analysis revealed that the serum total IgE level was weakly but significantly correlated with BMI (r = 0.1), waist circumference (r = 0.191), systolic blood pressure (r = 0.136), and triglyceride level (r = 0.148) (all P < 0.001; Table 2). The total IgE level was negatively correlated with the HDL cholesterol level (r = –0.067, P = 0.034). Total and allergen-specific IgE levels were positively correlated with FBG levels and HOMA-IR scores (all P < 0.05).

**Table 2:** Pearson’s correlation coefficients between serum immunoglobulin E (IgE) and metabolic parameters.

	Total IgE		House dust mite- specific IgE		Cockroach-specific IgE		Dog-specific IgE	
	R	P-Value	R	P-Value	R	P-Value	R	P-Value
Body mass index	**0.100**	<0.001	0.048	0.104	0.084	0.006	0.062	0.125
Waist Circumference	**0.191**	0.001	0.093	0.002	**0.156**	<0.001	**0.121**	<0.001
Systolic blood pressure	**0.136**	<0.001	0.033	0.327	**0.117**	<0.001	0.080	0.014
Fasting glucose	**0.130**	<0.001	0.086	0.002	**0.153**	<0.001	**0.141**	<0.001
Triglyceride	**0.148**	<0.001	**0.131**	<0.001	**0.134**	<0.001	0.090	0.004
HDL cholesterol	**−**0.067	0.034	**−**0.025	0.644	**−**0.092	0.006	**−**0.023	0.432
HOMA-IR	**0.105**	<0.001	0.062	0.034	**0.104**	0.003	0.085	0.005

HDL, high-density lipoprotein; HOMA-IR, homeostasis model assessment of insulin resistance.

**Figure 2:**
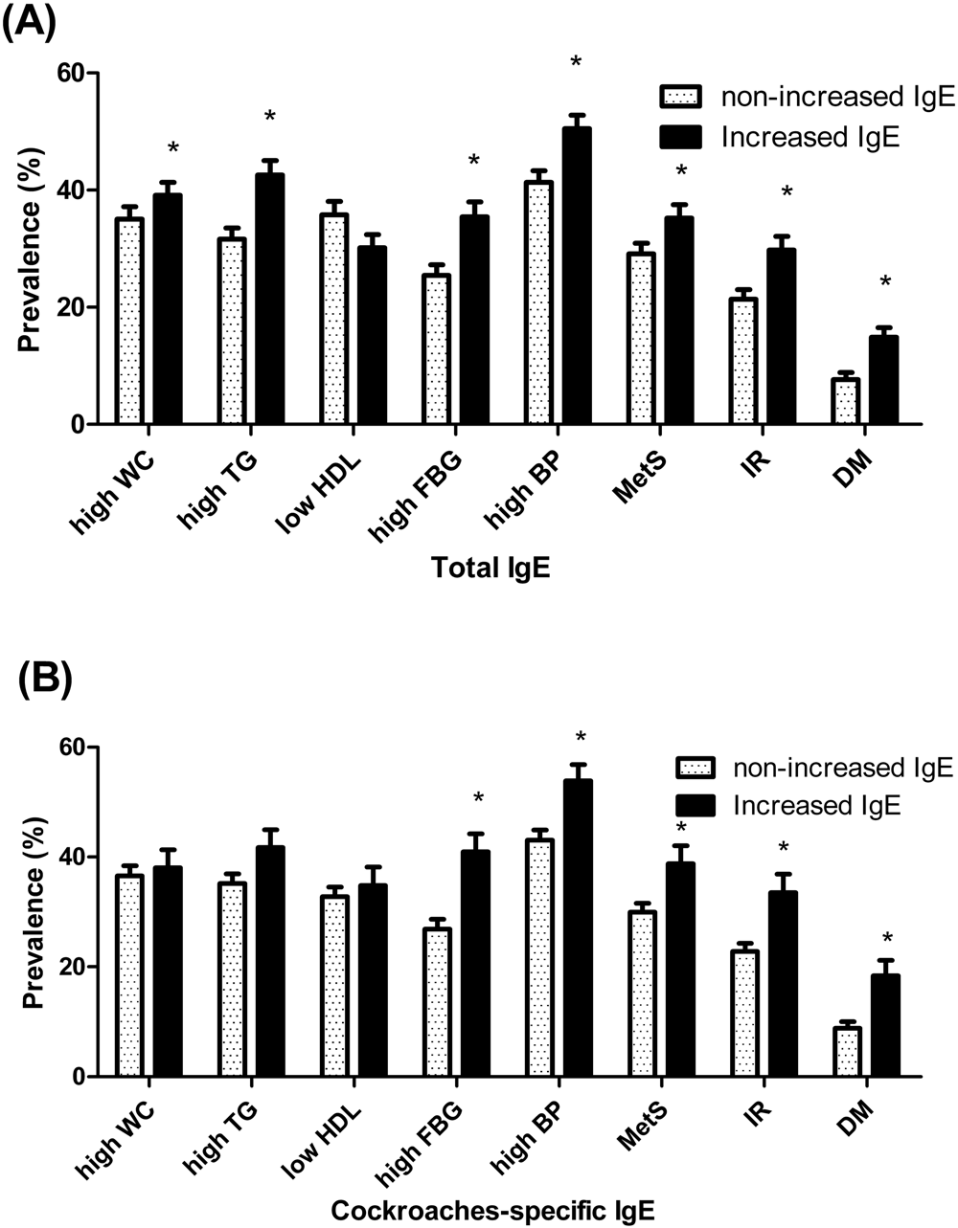
Differences in prevalence of metabolic syndrome/its components according to the “increased total IgE” (**A**) and cockroaches-specific IgE sensitization (**B**). High WC; high waist circumference, high TG; high triglycerides, low HDL; low high-density lipoprotein cholesterol, high BP; high blood pressure, MetS; metabolic syndrome, IR; insulin resistance, DM; diabetes mellitus. *P < 0.05.

The prevalences of hypertriglyceridemia (high TG), a high FBG level, elevated blood pressure (high BP), metabolic syndrome (MetS), and IR were significantly higher in the increased IgE group than in the non-increased IgE group (Fig. 2A). In terms of allergen-specific IgE levels, those sensitized to the cockroach had higher FBG levels and higher BP and more commonly exhibited MetS and IR (Fig. 2B).

### Risk of DM by IgE sensitization

Participants with increased total IgE were at increased risk of DM (OR 1.59; 95% CI, 1.06–2.37) after adjusting for age, sex, BMI, household income, educational level, and region of residence (Table 3, model 1). After further controlling for other confounding factors, including previous allergic disease, the association remained significant (OR 1.76; 95% CI, 1.18–2.61; Table 3, model 3). When we adjusted our full model for WBC counts, increased total IgE remained significantly associated with DM (OR 1.66; 95% CI, 1.10–2.51; Table 3, model 4).

**Table 3:** Unadjusted, adjusted OR and 95% CI of diabetes mellitus by increased Ig E and allergen sensitization.

	Total population			Non-allergy subjects		
	OR	95% CI	P-value	OR	95% CI	P-value
**Increased serum total Ig E**						
Unadjusted	**2.10**	**1.43–3.08**	<0.001	**2.38**	**1.61–3.52**	<0.001
Model 1	**1.59**	**1.06–2.37**	0.025	**1.76**	**1.15–2.69**	0.009
Model 2	**1.72**	**1.17–2.54**	0.006	**1.81**	**1.19–2.75**	0.006
Model 3	**1.76**	**1.18–2.61**	0.006	**1.81**	**1.19–2.75**	0.006
Model 4	**1.66**	**1.10–2.51**	0.016	**1.70**	**1.09–2.64**	0.018
**Sensitization to House dust mite**						
Unadjusted	1.34	0.88–2.06	0.177	**1.58**	**1.02–2.45**	0.039
Model 1	1.44	0.91–2.23	0.124	1.58	0.97–2.56	0.065
Model 2	1.57	0.99–2.49	0.056	**1.65**	**1.02–2.69**	0.044
Model 3	**1.63**	**1.03–2.59**	0.038	**1.65**	**1.01–2.69**	0.044
Model 4	1.51	0.94–2.43	0.091	1.54	0.94–2.53	0.090
Model 5	1.29	0.71–2.35	0.401	1.34	0.7–2.57	0.375
**Sensitization to cockroach**						
Unadjusted	**2.32**	**1.42–3.81**	<0.001	**2.53**	**1.50–4.28**	<0.001
Model 1	**2.20**	**1.34–3.62**	0.002	**2.37**	**1.41–3.98**	0.001
Model 2	**2.26**	**1.39–3.65**	0.001	**2.37**	**1.42–3.94**	0.001
Model 3	**2.27**	**1.40–3.66**	0.001	**2.37**	**1.42–3.94**	0.001
Model 4	**2.26**	**1.38–3.71**	0.001	**2.33**	**1.38–3.94**	0.002
Model 5	**2.16**	**1.21–3.84**	0.009	**2.25**	**1.21–4.18**	0.010
**Sensitization to dog**						
Unadjusted	1.18	0.44–3.22	0.740	1.67	0.59–4.70	0.334
Model 1	1.25	0.53–2.97	0.608	1.59	0.67–3.78	0.294
Model 2	1.31	0.53–3.23	0.554	1.65	0.68–4.03	0.268
Model 3	1.38	0.56–3.36	0.482	1.65	0.68–4.03	0.268
Model 4	1.21	0.51–2.83	0.668	1.51	0.65–3.51	0.336
Model 5	0.87	0.35–2.19	0.770	1.15	0.46–2.87	0.766

Model 1 is adjusted for age, sex, BMI, education, household income and region.

Model 2 is adjusted for model 1 plus smoking, alcohol drinking and regular exercise.

Model 3 is adjusted for model 2 plus allergic diseases.

Model 4 is adjusted for model 3 plus white blood cell counts.

Model 5 is adjusted for model 4 plus presence of total IgE sensitization.

Subjects sensitized to the house dust mite, compared with those without, had an OR of 1.63 (95% CI, 1.03–2.59) for DM after adjusting for age, sex, BMI, educational level, household income, region of residence, smoking status, alcohol consumption, exercise, and previous allergic disease. However, after additionally adjusting for WBC counts, the relationship was insignificant. Subjects sensitized to the cockroach also had an increased OR for DM after adjustment for various covariates (OR 2.27; 95% CI, 1.40–3.66; Table 3, model 3). The results did not change after adjusting WBC counts. Moreover, when we added “increased total IgE” and “sensitization of cockroach” as separate variables in the same multivariate model, sensitization of cockroach is an independent predictor of risk of DM (OR 2.16; 95% CI, 1.21–3.84; Table 3, model 5), regardless of total IgE. We performed additional analysis after excluding the subjects with previous allergic diseases. When subjects with previous allergic diseases were excluded, the results were similar compared to the total population (Table 3).

When poor glycemic control was defined as an HbA1c level >7%, sensitization to the cockroach was an independent risk factor for poor glycemic control in subjects with DM. Those exhibiting cockroach-specific IgE sensitization were at greater risk (OR, 7.36; 95% CI, 2.49–21.8, P < 0.001) of poor glycemic control. Further controlling for previous allergic disease did not attenuate the association between IgE sensitization and poor glycemic control (data not shown).

## Discussion

This is the first study to evaluate the association between serum IgE levels and DM in a general population. We found strong positive associations between total IgE, house dust mite and cockroach-specific IgE levels and DM, and these persisted after adjustment for many potential confounders, including lifestyle factors (alcohol consumption, smoking status, and regular exercise); socioeconomic status (income, educational level, and region of residence); and previous allergic disease. Moreover, cockroach-specific IgE was an independent risk factor for poor glycemic control in subjects with DM. We also found significant associations between metabolic syndrome and IgE sensitization. Our data suggest that IgE level may be an important independent risk factor for metabolic disease, especially DM, in Koreans.

Total IgE level is a biomarker closely associated with the inflammatory response to non-specific environmental factors as well as to reactions to specific allergens^2^. The serum IgE levels significantly increase in patients with coronary artery disease when compared to unaffected subjects^19^. IgE activates mast cells, which, experimentally, play a critical role in type 2 DM^1^. Genetic deficiency and pharmacological inactivation of mast cells prevented diet-induced type 2 DM in mice. A human epidemiological study has shown that patients with type 2 DM had increased levels of IgE and mast cell chymase^2^. The role of mast cells in diabetic nephropathy has been extensively reported^3^. Mast cells are uncommon in normal kidney tissue, but mast cell infiltration is a prominent and early response to renal injury. Mast cells trigger fibrosis and extracellular matrix accumulation in patients with diabetic nephropathy^20^. Therefore, it is possible that IgE participates directly in DM pathogenesis and in complications thereof.

However, it is equally possible that DM or high FBG levels trigger IgE sensitization. In mice with DM, high FBG levels impaired B-1 cell function; the immunoglobulin profile changed^21,22^. The numbers of B-1 cells decreased in patients with type 2 DM^21,22^. Gestational DM increased the risk of AD and allergen sensitization in early childhood^23^. Term infants of women with gestational DM were at a 7.6-fold increased risk of AD and a 5.9-fold increased risk of allergen sensitization. Prenatal exposure to high levels of glucose increased the risks of AD and sensitization in early life^23^. Mothers with gestational DM had higher levels of TNF-α, leptin, and visfatin and lower levels of adiponectin. Adiponectin attenuates allergic inflammation in murine models. Thus, it is possible that the changes in adipokine levels associated with gestational DM may affect immunological development in infancy^23^.

DM, obesity, and allergen sensitization share inflammatory pathways and mediators of immune responses. Increasing epidemiological evidence suggests that obesity increases the risks of asthma, atopic eczema, and allergic rhinitis. Obesity may trigger immunological changes, reducing antigen tolerance and skewing the immune system toward a T helper 2 (Th2) profile, which is associated with a cytokine release pattern increasing the risks of allergy and other immune-mediated diseases^24^. Previous studies have explored relationships between obesity and IgE sensitization^3^, but the data have been inconsistent. Vieira *et al*.^25^ found that the frequency of IgE-positivity in response to a balanced mixture of common aeroallergens was three-fold greater in obese than non-obese females. However, the total IgE concentration did not differ significantly between the groups. In the present study, central obesity, measured using waist circumference as a surrogate, was significantly (positively) correlated with total and all three allergen-specific IgE levels. We also found significant associations between metabolic syndrome and increased IgE.

Higher levels of allergic cockroach sensitization increased the risk of DM in the general population, and this association was stronger than that involving house dust mite sensitization. Cockroach extracts contain pepstatins, which are A-sensitive proteases activating the PAR-2 receptors; these, in turn, induce human eosinophil activation and degranulation. PAR-2 activation has been implicated in the potent allergenicity elicited by cockroaches^26^. PAR-2 contributes substantially to inflammatory and metabolic dysfunction, and PAR-2 antagonists inhibit diet-induced obesity, reverse IR, and glucose intolerance, and they beneficially modulate liver and pancreatic metabolic parameters^27^. Cockroach allergies are more common in infested areas. Low socioeconomic status has been independently associated with sensitization to cockroach allergens. We examined the effects of socioeconomic status on the association between IgE level and DM and found that neither household income nor educational level nor region of residence affected the observed associations. Sensitization of cockroach is an independent predictor of risk of DM regardless of total IgE. However, the question remains whether glucose intolerance can be improved by avoiding the sensitization of cockroaches.

A recent US population-based study^7^ showed that AD was associated with adult-onset diabetes (adjusted OR, 1.35; 95% CI, 1.13–1.62), reflecting higher levels of smoking and alcohol intake and a sedentary lifestyle^7^. In contrast, adult inpatients or outpatients with AD treated in a Danish national hospital were not at an increased risk of new-onset DM^10^. After adjustment for co-morbidities and medication use, the independent OR for DM decreased significantly (adjusted HR, 0.76; 95% CI, 0.68–0.83). However, in the present study, we found that increased IgE levels were associated with a rise in the prevalence of both DM (adjusted OR, 1.76; 95% CI, 1.18–2.61) and IR in a general population. IgE sensitization may explain the observed association between AD and the increased risk of DM.

B cells are emerging players in innate and adaptive immune responses associated with metabolic diseases, including obesity, type 2 DM and cardiovascular disease^21^. Immunoglobulin production is a prototypical function of B cells, and immunoglobulins also play an important role in inflammatory diseases including lupus, rheumatoid arthritis and atherosclerosis^22^. It was reported that decreased IgG and IgM, and increased IgA levels were independently related to the prevalence of type 2 DM^22^. But, no significant elevated IgE in patients with diabetes was observed in that study^22^. Although the reasons for this discrepancy with our results remain unclear, it may be due to the difference in the study population and the use of allergen-specific IgE. We have measured allergen-specific IgE as well as total IgE, and we found that cockroach specific IgE sensitization was significantly associated with DM. Mouse studies suggest that immature B cells in adults and neonatal B cells are more prone to IgE switching than mature B2 cells^28^. Immunoglobulin dysregulation might contribute to the development of DM. Further studies are needed to explore the causality and exact mechanisms of immunoglobulins in subjects with DM.

There are limitations to our study. First, this was a cross-sectional study, and, therefore, the association found between IgE sensitization and DM may not be causal. Second, we had no data on immune elements other than IgE. A previous study indicated that markers of inflammation (such as WBC counts) were associated with the development of DM in middle-aged adults^29^. WBC as a significant inflammatory factor is both accessible and affordable. Indeed, there was a significant difference of WBC counts between DM and non-DM subjects. So, we added WBC counts into model 4 of multivariate analysis. We found that increased total IgE and sensitization to cockroach remained significantly associated with DM after adjustment for WBC counts. Third, the history of steroid use was not available in this study. Fourth, the seasons of sample collection could not be discerned. According to previous studies conducted in Korea, not only pollens but also house dust mites demonstrate seasonal variation at indoor concentrations^30^. However, the rate of sensitization to cockroaches was similar for all seasons, except in summer^30^. Seasonal changes in sensitization to house dust mites have occurred in populations with allergies. We performed additional analysis after excluding the subjects with previous allergic diseases. The results were similar compared to the total population. Lastly, we could not differentiate type 1 or gestational from type 2 diabetes from the KNHANES data set. This study was conducted for adults over 30 years of age and prevalence of type 1 or gestational diabetes is relatively low. The prevalence of type 1 diabetes during 2011–2013 was about 0.02% of the total population in Korea^31,32^. However, we could not exclude the possibility that participants with type 1 or gestational diabetes were included in this study and the association of IgE with prevalence of diabetes differed according to diabetes type^33^.

In conclusion, we found that, independent of any previous allergic disease, an increased IgE level and sensitization to the cockroach were risk factors for DM. Sensitization to the cockroach was significantly associated with uncontrolled DM either before or after adjustment. As we used data from a nationally representative sample of the population and adjusted for multiple confounders, our results support the hypothesis that increased serum IgE levels are associated with DM in the general population.
